# “It has to work for us”: A qualitative study exploring how lived experience engagement reframed development of a mental health module within a Spinal Cord Injury Self-Maintenance Tool

**DOI:** 10.1038/s41393-026-01171-8

**Published:** 2026-02-03

**Authors:** John Bourke, Ashley Craig, Danielle Sandalic, Mohit Arora, K. Anne Sinnott Jerram, James W. Middleton

**Affiliations:** 1 John Walsh Centre for Rehabilitation Research, Northern Sydney Local Health District, St Leonards, NSW Australia; 2 The Kolling Institute, Faculty of Medicine and Health, The University of Sydney, Sydney, NSW Australia; 3 Spinal Cord Injuries Unit, Royal North Shore Hospital, Northern Sydney Local Health District, St Leonards, NSW Australia; 4 Spinal Outreach Service, Royal Rehab Group, Ryde, NSW Australia

**Keywords:** Rehabilitation, Quality of life

## Abstract

**Introduction:**

People with spinal cord injury can experience various psychosocial challenges when adjusting to their injury. It is essential they have access to evidence-based resources that can enhance their adjustment. During the development of the Spinal Cord Injury Health Maintenance Tool preliminary lived experience feedback highlighted the need to include an additional module on mental health.

**Study design:**

Qualitative study.

**Objectives:**

To co-design a new Mental Health module with people with spinal cord injury during the digitisation of the Spinal Cord Injury Health Maintenance Tool.

**Setting:**

Community in Sydney, Australia.

**Methods:**

Two focus groups involving participants with lived experience of spinal cord injury (n=5 and n=4) were combined with a one-off expert review process.

**Results:**

Five key informants engaged in the collaboration. Four key themes summarised how this second round of lived experience progressed the mental health resource: 1) *Mental health is front and centre* highlighted the necessity of including mental health in any self-management plan for spinal cord injury; 2) *Striking the right balance* acknowledged the serious impact of mental health issues but without undue negativity; 3) *The glass is half full* emphasised the need for a greater focus on a strengths-based approach; and 4) *Variety’s the spice of life* required the Mental Health module to respond to individual circumstances.

**Conclusions:**

Lived experience feedback ensured that the tone, scope and content of this module were revised to place greater emphasis on a strengths-based orientation, while still acknowledging the serious and severe outcomes associated with compromised mental health.

## Introduction

Spinal cord injury (SCI) may result from either a traumatic injury or a non-traumatic disease or disorder and can have major impacts on health, functioning, participation and quality of life [1]. Furthermore, the onset of a SCI is often sudden and can lead to a range of complex biopsychosocial consequences that challenge a person’s personal resources, which may compromise mental health and wellbeing [2]. With the prevalence of SCI growing over the past 30 years [3], there is an increasing pressure on primary care services to support those with SCI and manage their complex secondary health complications, which is compounded by reduced lengths of stay in post-acute rehabilitation units and a lack of specific SCI knowledge and skills within the primary care sector [4–6]. Consequently, there is an increasing need for resources enabling people with SCI and primary health care services to manage their health in community settings [7, 8].

In addition, there is an increasing need to ensure SCI rehabilitation interventions are co-designed with people who have the lived experience of SCI. The use of participatory methods in health research has attracted increasing attention and enthusiasm in recent decades [9–11]. Co-design, which has its origins in Scandinavian ‘co-operative’ or ‘participatory’ design [12, 13] involves partnership with people with lived experience in one or more stages of the research process (although a substantive approach may encompass all stages) [12]. Moreover, co-design reflects a renewed focus on collaborative, person-centred models of healthcare provision and a greater involvement of patients and community members in health research [14]. To address these dual needs of creating and co-designing self-management resources for people with SCI, our team co-designed a Spinal Cord Injury Health Maintenance Tool (SCI-HMT) between 2018-2023 [15]. The SCI-HMT is a digital and hardcopy resource to support the self-management of the most common secondary conditions associated with SCI, which initially covered the topics of bladder, bowel, skin, pain and autonomic dysreflexia.

During the iterative co-design development process of the SCI-HMT, participants with the lived experience, as well as primary healthcare professionals, made it clear that addressing mental health needed to be a fundamental priority [15]. Indeed, there is an increasing need to design more positive, process focused resources to assist individuals to self-manage mental health and adjustment following SCI [16]. For example, our team’s previous research has identified that following a SCI, as many as 70% of people do not meet the criteria for a clinically diagnosable mental health disorder, however, are still likely to experience psychological and emotional distress that may be exacerbated by catastrophising styles of thinking [2, 17]. Co-designed resources to facilitate the development of self-efficacy and psychological resilience would be very beneficial for this cohort [16].

**Fig. 1:**
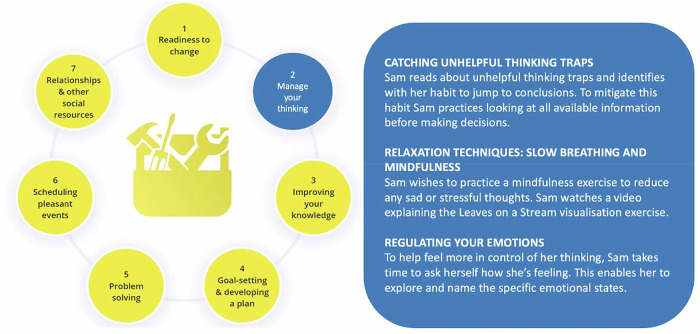
The mental health toolbox includes seven sections that have an evidence-base as an effective psychological therapy. Each section includes a range of strategies to assist people develop a personalised program. Figure 1 shows a fictitious example of how a person might use the “manage your thinking” section.

On the collective basis of the previous work, an additional module for the SCI-HMT was co-designed with consumers - The Mental Health and Wellbeing Module (MHM). The MHM is designed to improve self-management and develop resilience and strengthen a sense of self-efficacy to promote positive adjustment. It includes a self-management Toolbox (see Fig. 1 for an example), featuring a variety of strategies and skills that are evidence-based and recognised as an effective psychological therapy [18, 19]. The MHM was designed to address: 1) the recognised need for interventions to address “subclinical” cognitive and psychological aspects of adjustment post-SCI, thus enabling positive adjustment and resilience [16] (especially given the experience of loss and grief following SCI), and 2) the absence of evidence-based, consumer-informed, best-practice resources for people with SCI to optimise their self-management.

The aim of this paper is to describe how lived experience engagement on the MHM draft complemented and built upon health professionals’ knowledge to refine the scope, tone, and content of the MHM; and to ensure the final iteration was accessible, usable, and practically applicable to support the self-care of people with SCI.

## Methods

The methodological approach of this study was underpinned by pragmatism which asserts that the research questions should address the concrete problems people face in everyday life in order to provide positive social action [20, 21]. Pragmatism moves the question away from metaphysical debates to focus on the immediate purpose of the research [20]. As mentioned above, the pragmatic approach taken in this study was to ensure the MHM was practically applicable to support the relevant, everyday self-care of people with SCI.

Regarding co-design, preliminary engagement with an expert informant group of individuals with the lived experience of SCI highlighted the need to include a MHM within the SCI-HMT. In response, a draft MHM was developed by a team of academic health professionals who specialise in psychosocial adjustment following SCI (AC, DS, MA, JWM). Content was informed by research evidence, including psychosocial best-practice guidelines [16, 22]. The original expert informant group of individuals with the lived experience of SCI was invited back to review the MHM and to discuss results from a focus group.

The process of lived experience involvement was reported using the Guidance for Reporting Involvement of Patients and the Public (GRIPP2) Short Form (see Table 1) and this project received ethical approval from the Northern Sydney Local Health District Human Research Ethics Committee, reference 2019/ETH13961. Written informed consent was obtained from all participants. All methods were performed in accordance with the ethical guidelines and regulations.

**Table 1:** GRIPP2 Short form^a^.

Section & Topic	Item	Details	Pg. no.
1. Aim	Report the aim of PPI in the study	To describe how engaging people with the lived experience of SCI could refine the scope, tone, and content of the MHM	Pg. 2
2. Methods	Provide a clear description of the methods used for PPI in the study	An expert informant group of individuals with the lived experience of SCI reviewed then discussed MHM via two focus groups.	Pg. 2
3. Study results	Outcomes—Report the results of PPI in the study, including both positive and negative outcomes	Four themes: Mental health is front and centre; Striking the right balance; The glass is half full and Variety’s the spice of life were interpreted to summarise how the engagement of people with the lived experience focused more emphasis on strengths-based orientation without dismissing acknowledgement of the serious and severe outcomes of MH.	Pg. 3
4. Discussion and conclusions	Outcomes—Comment on the extent to which PPI influenced the study overall. Describe positive and negative effects	Lived experience engagement ensured the MHM was evidenced based, but also and useable, acceptable and appropriate for people with SCI.	Pg. 5
5. Reflections/critical perspective	Comment critically on the study, reflecting on the things that went well and those that did not, so others can learn from this experience	Engaging lived experience perspectives was instrumental in ensuring the MHM addressed authentic concerns and was not just constructed by people without such direct experience, however well- intentioned such people might be.	Pg. 5

^a^Staniszewska, S., Brett, J., Simera, I., Seers, K., Mockford, C., Goodlad, S., … Tysall, C. (2017). GRIPP2 reporting checklists: tools to improve reporting of patient and public involvement in research. BMJ, 358, j3453.doi:10.1136/bmj.j3453.

### Participant recruitment

Participants with the lived experience of SCI from the original expert informant group (who initially recommended a MHM be developed as part of the SCI-HMT) were invited back to participate in a follow-up focus group to review the first draft of the MHM.

### Data collection

Two online focus groups with participants who have the lived experience of SCI (n = 5 and n = 4 respectively) were conducted. In each, participants were asked to review the content, tone, style and structure of the MHM in terms of its usefulness and practical relevance to people with lived experience of SCI. Participants were sent a pdf version of the MHM one week before the focus group. The first focus group was facilitated by KASJ using Microsoft Teams with five participants along with MA and JWM. The second focus group was also facilitated by KASJ using Microsoft Teams with four participants primarily attending via Microsoft Teams. Each focus group was approximately 1 h in duration. The first draft version of the MHM was made available to the participants prior to the second focus group. An interview guide was used to guide discussion (see supplementary file 1).

### Data analysis

Focus groups were audio recorded and transcribed verbatim with the information collected subjected to reflexive thematic analysis to actively interpret codes, patterns and themes relative to the research question [23]. Transcript data was read and re-read by two members of the research team independently (KASJ, MA), who interpreted codes in the first instance (items of data reflecting a single concept). These codes were then arranged into broader patterns, before being presented to the whole research team for deliberation and development of themes (an arrangement of data reflecting a broad idea). In addition, the MHM content and data were reviewed by an SCI lived-experience researcher with a background in psychology and disability studies (JB).

### Research team reflexivity

Research team reflexivity (i.e., considering and acknowledging the values, skills and experience that every researcher brings to a research study) was enacted by examining and reflecting on the individual and collective influences of the research team via email discussions. For example, the focus group was facilitated by one author (KASJ) who has >20 years as an allied health professional and experience of qualitative, co-design research with SCI populations. Additional members of the team (AC, DS, MA and JWM) are specialists in psychosocial adjustment following SCI while JB has >20 years lived experience of high-level SCI as well as a PhD in Health Sciences. Collective reflection of the team highlighted the critical importance on including lived experience perspectives when developing resources such as the MHM.

## Results

Five people participated, three female and two males (individual data not reported to avoid identification). Participants had a range of SCI characteristics (incomplete/complete; tetraplegia/paraplegia) as defined in the ASIA/ISCoS International Standards for Neurological Classification of Spinal Cord Injury (ISNCSCI). Through this collaborative co-design process, the MHM was fittingly modified so that the content was comprehensible, suitable for people’s different circumstances, and maintained a strengths-based orientation while recognising that serious mental health challenges can still arise [15]. Four themes were interpreted to summarise how lived experience improved the MHM. Each theme is described below with illustrative quotes.

### Theme one: mental health is front and centre

The first theme described participants’ collective agreement that having a module focusing on mental health was most certainly a non-negotiable necessity for a community self-management resource for people with SCI. This theme was evidenced through various references to mental health being the foundation on which other self-management strategies can be applied. As one participant said: *yes the* [MH] *tool box is good because it allows mental health to sit in front of all other topics* [FG001]. In a similar vein, another participant highlighted how addressing mental health can increase the readiness of people to engage in other SCI-HMT topics: *because all other* [HMT] *topics like bladder care for example are further back in one’s mind - compared with mental health* [FG004].

Participants reported how having mental health front and centre served to normalise the significant challenge that a SCI presents to a person’s coping resources. Just knowing that many people experience significant mental health challenges was helpful in itself, as one participant reflected: *even just saying it’s* (mental health) *is normal is a great help… I think definitely mention it* (mental health) *because then someone really will think, oh phew. I’m not abnormal after all* [FG004]. Indeed, having a resource that acknowledged how MH challenges are a very real part of the adjustment process following SCI was welcomed by participants. For example, one participant highlighted how following a SCI, people can question whether they have the capacity to go on:

“*It is really nice and simple and given that a lot of people – maybe most newly injured people – like I did myself – just want to stop living after their injury – I think the toolbox is a sort of relief to read” [FG003]*.

In summary, theme one underscored that mental health was indeed a priority issue. However, participants were quick to remind researchers that it was important to promote the importance of mental health for functioning and wellbeing without overwhelming readers with too much detail.

### Theme two: striking the right balance

The second theme describes participants’ desire for the MHM to strike the right balance of acknowledging the serious impact of mental health issues but without undue negativity. For example, while the honest framing of mental health issues was considered positive: *“I like it. It is to the point – quite blunt”* [FG004], concern was expressed that people with SCI who might read this content may already be feeling ‘vulnerable’ and be susceptible to an overemphasis on negative outcomes. As one participant said: *“things like mood fluctuate enormously, especially in the early days – enormous variation in every given day – even an hour”* [FG003]. Participants indicated that in the original “academic” draft, the complex language, theoretical constructs, and style placed too much emphasis on the negative aspects of mental health disorders and management, which could detract from its value:

*“Well I think it is really useful and might allow people to sort out what the greatest concern is. But you have to be careful that if I wasn’t depressed, I might be after looking at this module*.” [FG003]

Participants were quick to emphasise that the toolkit should never be seen as a substitute for professional help. Since deteriorating mental health can have very serious consequences, participants consistently stressed the fine line which exists between trying to self-manage one’s mental health and knowing when (and how) to seek professional support - a decision which can be further clouded by deteriorating mental health. As one participant said,

*“We can’t take away from the fact that people, like, if they are suicidal, if they are having, like depression and anxiety, they really need to seek professional support*.” [FG002]

Participants also highlighted that no matter how well a self-management resource may be designed, and what information it may communicate, there is essentially (particularly in serious cases) no substitute for professional support. For example, one participant remarked:

*“Perhaps that’s when it’s, you refer on to services… because I think that there’s a there’s maybe a, a level of support that that person requires that maybe that the tool won’t be up to address* [everything] *and help is needed*.” [FG001]

In summary, theme two described how the MHM needed to strike a delicate balance between highlighting the potential to experience serious mental health issues while not overly stressing the negative aspects of adjustment following and SCI. This can be helped through a positive orientation, which is examined below.

### Theme three: the glass is half full

The third theme emphasised the need for the MHM to focus on positive aspects of the adjustment process. While feedback suggested some negative mental health outcomes need to be mentioned, participants felt several negative adjectives could be removed (for example *unhappy, miserable*) to reduce the negativity without lessening the seriousness of the message. Participants felt that the information within the MHM could describe both the challenging realities of adjustment, as well as emphasise the positive outcomes implicit in the adjustment process. For example, one participant said:

*“It’s not nice to have to go through bladder issues and bowel issues where you know this grief that you’re going through is totally normal* (and) *through grieving, through process of acceptance, there is light at the end of the tunnel*.” [FG002].

A more positive framing was achieved through including a simplified description of an evidence-based adjustment model based on the SCIAM [19] (which emphasises the influential role of personal appraisal and the power of a person to direct their mindset and behaviours). The original description was too complex, as one participant said:

*“The words in the diagram, this SCI adjustment model. There’s a lot of very long words there that could be expressed more simply*.” [FG004]

In response, the simplified description of the SCIAM was intended to fulfil two key functions: 1) to ensure that the message was understandable to a wide range of potential readers with different levels of health literacy, and 2) emphasising that a person still has control over their adjustment process and outcomes following a SCI. Often this sense of control may not be readily apparent, and may depend on a person’s readiness to change, as one person remarked:

*“People are only going to access the resource if they are ready to acknowledge that they may be suffering from mental health illness and wanting to change their circumstances to be more informed and improve their mental health”* [FG002]

In summary, theme three underlined the need to acknowledge that positive outcomes were possible following a SCI, and that the MHM needed to have resources to assist people to understand aspects of adjustment within their control, which were applicable to a variety of people. This leads to our last theme, which highlights the need to create a module which is sensitive to individual circumstances.

### Theme four: variety’s the spice of life

The fourth theme highlighted the need for the MHM to be sensitive to individual circumstances and to not imply certain outcomes would inevitably apply to everybody, as one participant said:

*“The thing is every person with a spinal cord injury is just that – one person with a spinal cord injury”* [FG003]

For this reason, words like ‘*will’* were replaced with ‘*might’* or ‘*can’*. For example, *If you do not resolve your grief, you* ***will**** become emotionally drained, at risk of feeling overwhelmed sad, angry* ***and**** worried* was changed to *If you do not resolve your grief, you* ***may**** become emotionally drained, at risk of feeling overwhelmed, sad, angry* ***or**** worried*.

At first glance, this may seem merely semantic tinkering. However, the potential for unintended and implicit negative messages to be passed on through the tone of language cannot be underestimated. For example, while many people do face significant adjustment challenges following SCI – not everyone does. It is easy to assume everyone will be susceptible to the same contributing factors or outcomes, as one participant said:

*“I wouldn’t generalise to say that everyone that has a spinal cord injury has that* [mental health challenges]*, but obviously there are a lot of people that do*.” [FG001]

Further efforts to highlight the variety of lived experience were adopted. These included inserting lived experience quotes as examples of positive outcomes, and the addition of several interactive activities in the Toolbox designed to help illustrate how people can develop unique strategies to reflect their circumstances (see Fig. 1 for worked example of somebody using the skills in step 2 of the Toolbox – Manage your thinking).

## Discussion

There is increasing recognition that partnering with people with lived experience increases the relevance of research priorities and outcomes [24, 25] and raises the quality of interpretation and knowledge translation [26] as it is more reflective of lived reality and can bridge the gap between research and practice [24, 27, 28]. Given the increasing pressure on primary care services for supporting health promotion and management in people with SCI, self-management resources must be as useable and effective as possible for the populations for which they are designed.

Neglecting to include lived experience perspectives, defined as the direct, first-hand accounts of certain experiences [29], risks having resources developed according to representations constructed by people without such direct experience, however, benevolent and well-intentioned such people might be. In the instance of designing the SCI-HMT (and the MHM in particular), engaging with people with lived experience of SCI helped to ensure these resources are both evidence-based and practically applicable to the community they are designed to serve.

In this paper, we have reported on how a process of lived experience engagement was central to improving the development of a MHM within the SCI-HMT. Involvement of individuals with lived experience enabled the tone, scope and content of this module to accurately reflect the reality of life with SCI. Specifically, lived experience engagement highlighted the need to have mental health positioned at the front and centre when developing the SCI-HMT. This reflects research which highlights that people with SCI can indeed experience a variety of psychosocial challenges when adjusting to their injury. For example a loss of identity and control, relationship stresses, social discrimination and reduced employment prospects, which can manifest in up to 20-30% of people with SCI as depression or anxiety disorders [2, 16].

Lived experience input provided a clear mandate for having a positive, strengths-based approach. With the management of cognitive and psychological conditions following SCI often tending to focus on the medical aspects of care, there is a critical need for positive, process orientated mental health tools to promote resources for people to appraise their SCI as something that is manageable. The nuanced feedback from those with a lived experience confirmed the need to acknowledge the potential for many serious mental health challenges to occur following SCI, however, without emphasising undue negativity. This strengths-based approach reflects the findings of a recent narrative review on the research surrounding mental health and SCI, which concluded that existing mental health guidelines concerning SCI too often neglect positive processes of adjustment [16].

A strengths-based orientation to the MHM was operationalised through the development of several strategies and activities in the self-management ‘Toolbox’. The Toolbox includes seven strategies, each focused on a particular set of skills that had an evidence-base as an effective psychological strategy. The intention of the toolbox is to assist readers to develop a personalised program acknowledging that the process of adjustment is dynamic, non-linear and an individualised process. Figure 1 outlines each of the seven strategies, with a fictious example of how a person might use the specific skills within the ‘manage your thinking’ strategy.

While up to 30% of people may develop a clinically diagnosable mental health condition following an SCI and require targeted clinical treatment, many more still require resources to address and help management of “non pathological” adjustment following SCI. A recent narrative review highlighted that “understanding non-clinical cognitive and psychological aspects of adjustment post-SCI is paramount and that the application of this knowledge to the formulation of adjustment-enhancing interventions is crucial” [16] (p. 1997). Users of the Toolbox are directed to different cognitive processes and encouraged to develop habits conducive to positive appraisal of their circumstances. Hence, outcomes are individualised and unique to a person’s environment and personal circumstances.

The Mental Health and Wellbeing module (as part of the overall SCI-HMT) is publicly available as a digital and hardcopy resource (see: https://healthmaintenancetool.com/your-health/mental-health/your-mental-health/). The initial launch of the SCI-HMT was informed by co-design work, which mapped lived experience perspectives to the domains of the Consolidated Framework of Implementation Research [30] for planning implementation of the SCI-HMT. The next critical step in advancing this work is to rigorously co-design and evaluate implementation strategies to best introduce and use the MHM in regular rehabilitation care, ensuring the sustainable adoption and impact of the MHM for people with SCI.

### Limitations

This research has several limitations which should be considered when interpreting the findings. Firstly, our small sample size may limit the diversity of perspectives recorded. It is very possible that recruiting more participants over a more sustained data capture window would have produced more insights. Secondly, our research may not fully reflect the experiences of people with SCI in other settings or cultures. Lastly, this research is not a description of a fully implemented resource where the real-world impact of psychosocial outcomes were measured. Addressing these areas in future research will help to ensure the MHM will be effective in addressing a range of implementation, clinical and individual-level base outcomes.

In conclusion, adopting a genuine, co-design process involving expert informants with lived experience of SCI helped to refine a mental health resource within a SCI health maintenance tool. Lived experience engagement ensured this module described the significance of mental health issues following SCI, but without unnecessarily overwhelming readers. Engagement ensured the module was clear about its scope, providing advice for seeking professional support when necessary. Lastly, engagement ensured the inclusion of an interactive mental health toolbox with a range of evidence-based strategies and activities which could be adapted to diverse individual circumstances.

## Supplementary information

Supplementary information

## Data Availability

The datasets generated during and/or analysed during the current study are available from the corresponding author on reasonable request.
