# Correction: “It has to work for us”: A qualitative study exploring how lived experience engagement reframed development of a mental health module within a Spinal Cord Injury Self-Maintenance Tool

**DOI:** 10.1038/s41393-026-01188-z

**Published:** 2026-03-13

**Authors:** John Bourke, Ashley Craig, Danielle Sandalic, Mohit Arora, K. Anne Sinnott Jerram, James W. Middleton

**Affiliations:** 1 John Walsh Centre for Rehabilitation Research, Northern Sydney Local Health District, St Leonards, NSW Australia; 2 The Kolling Institute, Faculty of Medicine and Health, The University of Sydney, Sydney, NSW Australia; 3 Spinal Cord Injuries Unit, Royal North Shore Hospital, Northern Sydney Local Health District, St Leonards, NSW Australia; 4 Spinal Outreach Service, Royal Rehab Group, Ryde, NSW Australia

**Keywords:** Rehabilitation, Quality of life

Correction to: *Spinal Cord* 10.1038/s41393-026-01171-8, published online 03 February 2026

The original online version of this article was revised to correct participant numbers, update methodological descriptions and ensure accuracy in page references and terminology throughout. Revisions also included corrections to Table 1 (GRIPP2 Short Form), specifically updating page number references to reflect the correct pagination. In addition, targeted adjustments were made in the reference list: References 14 and 30 required correction of the author name formatting.

