# Supplementary material for: “It has to work for us”: A qualitative study exploring how lived experience engagement reframed development of a mental health module within a Spinal Cord Injury Self-Maintenance Tool
Source: Spinal Cord. 2026 Feb 3;64(4):346–51. doi: 10.1038/s41393-026-01171-8 (PMC13065473; doi:10.1038/s41393-026-01171-8)
Supplement: Supplementary file 1 — Supplementary information [file 41393_2026_1171_MOESM1_ESM.pdf]

SCI Wellness Project
Semi-structured interviews for the MH module

Interviewer schedule

Thank you for your interest to participate in phase 2 of the SCI Wellness Project involving Digitalisation of the Health Maintenance Tool for people with spinal cord injury. My name is ___________ (Interviewer), and I am _______________ (Interviewer designation) at the John Walsh Centre for Rehabilitation Research based at The University of Sydney.

The aim of this project is to deliver a digital version of the health maintenance tool to assist people living with SCI in self-managing their spinal needs over a lifetime. We will be asking you some questions about your health and wellbeing.

Dear ________, before we start the interview, would you like to ask me anything about this project or do you have any concerns?

Before beginning, I would like to ask your permission to record today's interview to ensure accuracy in transcribing your valuable information. Your identity is XXXX and XXXX.

**_For internal Use:_**		
Is the recorder on and working?	☐ Yes	☐ No

Recording:
This is Semi-structured one-on-one interviewing for the SCI Wellness Project.

My name is ____________ *and today on ___ of _______ 2020 at ____: ____ hrs, I am interviewing*

_____ (Initials of participant) with participant ID _______.

I. Health Behaviour (Attitude and Perceived Needs)

1. What motivates you to **PRACTISE** or **INITIATE** thoughts and behaviours that promote positive mental health and/or emotional wellbeing? For example, feeling good in yourself, connecting with others or keeping physically active.

2. There are number of ways people use to manage their mental and emotional health. Describe how you ensure that you stay mentally and emotionally well. Please check any of the options below that you perform on daily basis:
    a. Having a positive or helpful attitude
    b. Socialising
    c. Doing things, I enjoy regularly
    d. Connecting with family, friends or significant others
    e. Maintaining close personal relationships
    f. Developing effective ways of coping
    g. Exercising regularly
    h. Eating well
    i. Managing my stress levels
    j. Using relaxation techniques, such as, meditating, mindfulness and visualisation
    k. Listening to music
    l. Ensuring that you get enough sleep.
    m. Helping others.
    n. Expressing gratitude

o. Building self-esteem and confidence
p. Changing any negative thinking
q. Avoiding risk-taking behaviours, such as using too much alcohol or drugs
r. Getting professional help if you need it
s. Becoming involved in sports, recreational activities and/or hobbies
t. Having a healthy sexual/ intimate relationship
u. Other, please specify……………………….……..

3. From those you have selected from the above list, can you tell us more about the top three strategies that you find most helpful.

4. Now I would like to explore these areas further in relation to your knowledge and confidence to self-manage

   A. Mood and Feelings
   - ✓ How will you know if you have a problem with your mental health, such as depressed mood, elevated anxiety/stress, or drug and alcohol misuse? Can you tell the difference between feelings of depression, grief/sadness and worry/anxiety?
   - ✓ Have you been able to manage the above problems?
   - ✓ When would you seek professional help?
   - ✓ Where would you go to get help if you need it?

   B. Developing effective coping skills
   - ✓ What have you found are effective ways of coping with unexpected changes and challenges in your life?
   - ✓ What can you do to improve your sense of control (that is, self-efficacy beliefs) and perceived 'manageability' of difficult or stressful situations?
   - ✓ What do you understand you need to do to build resilience?

   C. Maintaining social relationships
   - ✓ How do you stay connected with family, friends or significant others? If you are connected, how often do you do this?
   - ✓ Do you receive any peer support from other people with spinal cord injury?
   - ✓ Do social barriers cause you to feel depressed or anxious?

   D. Are there other factors that are important for maintaining Mental Health and Emotional Wellbeing?
   - ✓ What about "*Maintaining healthy sleep behaviour*" Is your mental health negatively affected by poor sleep? How do you manage your sleep?
   - ✓ What about "*Managing my pain*" Is your mental health affected by ongoing pain? If so, do your strategies to improve your mental health also help your levels of pain?

II. Follow-ups

5. How often do you have an appointment with your GP or other health professional? Do you discuss issues relating to your mental health and emotional wellbeing?

III. Source and accessibility of Information

6. What information do you find most useful in managing your mental health and emotional wellbeing? For example, medical advice, information from Consumer organisation, online web resource, and so on.

7. Where do you obtain this information?

IV. A new tool and accessibility

8. What do you expect in a new tool to support decision-making about your mental health, emotional wellbeing, adjustment and coping needs after spinal cord injury?

V. Knowledge about other tools

9. Are you aware of an app or a guide that people with SCI use for maintaining their mental health and emotional wellbeing?

VI. Facilitators, Challenges and Barriers

10. Knowing what do you now about your spinal cord injury, if you had the chance what would you tell your younger self to help to better manage your mental health and emotional wellbeing?

11. In your opinion, what challenges and barriers that affect mental health and wellbeing do people with SCI face being in our society?
